# Anti-Inflammation Effect of Small Molecule Oligopeptides Prepared from *Panax ginseng* C. A. Meyer in Rats

**DOI:** 10.3390/molecules24050858

**Published:** 2019-02-28

**Authors:** Meihong Xu, Qihe Chen, Rui Fan, Junbo Wang, Yong Li

**Affiliations:** Department of Nutrition and Food Hygiene, School of Public Health, Peking University, Beijing 100191, China; xumeihong@bjmu.edu.cn (M.X.); qiheyuntian@163.com (Q.C.); fanruirf@bjmu.edu.cn (R.F.); Mrwang4j@163.com (J.W.)

**Keywords:** *Panax ginseng*, oligopeptide, inflammation, cotton pellet-induced, dextran

## Abstract

The present study was designed to investigate the anti-inflammatory effects of ginseng oligopeptides (GOPs). For the anti-inflammatory activity, dextran-induced paw edema and granuloma models were used in Sprague-Dawley rats (180–200 g, 12 weeks old, *n* = 10). Rats were treated orally with GOPs (0, 62.5, 125, 250 and 500 mg/kg) for prophylaxis. In the granuloma model, the levels of NO, Tumor necrosis factor-α (TNF-α), interleukin IL-β, and interleukin IL-10 in serum were evaluated. In addition, in the edema model, the level of TNF-α, prostaglandin E2 (PGE2), Leukotriene D4 (LTD4), and the platelet activating factor (RAF) in paw tissue were detected. PCR assessed the effect of GOPs on the expression of MAPK and NF-κB. The results showed that oral administration of GOPs inhibited inflammation caused by cotton pellet and dextran. GOPs significantly inhibited the edema formation via MAPK and NF-κB. These findings suggested that GOPs have a beneficial effect on acute and chronic inflammation, and the mechanism possibly mediated by inhibiting gene expression involved in inflammation and downregulating inflammatory mediators.

## 1. Introduction

As a physiological process, the inflammation response is triggered by activating some mechanisms. It causes complex changes in humoral and cellular components after tissue injury or infectious agents, vascular permeability, cellular migration, and the release of cytokines and free radicals [1,2]. Inflammation not only results in tissue edema, pain and damage, but also may contribute to chronic disease development, such as obesity, diabetes, psoriasis, pharyngitis, arthritis, and cancer [3,4]. In order to regulate the inflammation responses and its effects, conventional drugs are used, including non-steroidal anti-inflammatory drugs and glucocorticoids [5]. However, numerous adverse effects have been reported about these drugs [6]. Thus, the focus of anti-inflammation is moving toward alternative agents such as natural products, bioactive molecules, and functional foods [7,8,9].

*Panax ginseng* C. A. Meyer, which is more than a traditional Chinese medicinal herb, has been used as food for several years all around the world. Numerous functionality studies have been conducted on the whole ginseng root, and extensive pharmacological functions of ginseng has been reported, including anti-tumor, antioxidant, immuno-regulation, and metabolic normalized [10,11,12]. Moreover, currently, various bioactive components of ginseng were extracted and discussed, such as ginsenosides, polysaccharides, peptides, polyacetylenes, and phenolic compounds. Today, ginseng oligopeptides (GOPs) are considered a kind of nutrient with high bioavailability and absorption features, which was extracted from ginseng protein. Moreover, it has been realized as a bioactive component, based on its potential benefit for health, including anti-fatigue, immune modulation, glycemic control, and liver protection [13,14,15,16]. All these studies showed that GOPs may have a great anti-inflammatory potential. Therefore, a scientific study to identify the underlying mechanisms of the anti-inflammatory properties of GOPs was carried out in rats.

## 2. Results

### 2.1. Effects of GOPs on Cotton Pellet-Induced Granuloma 

**Figure 1:**
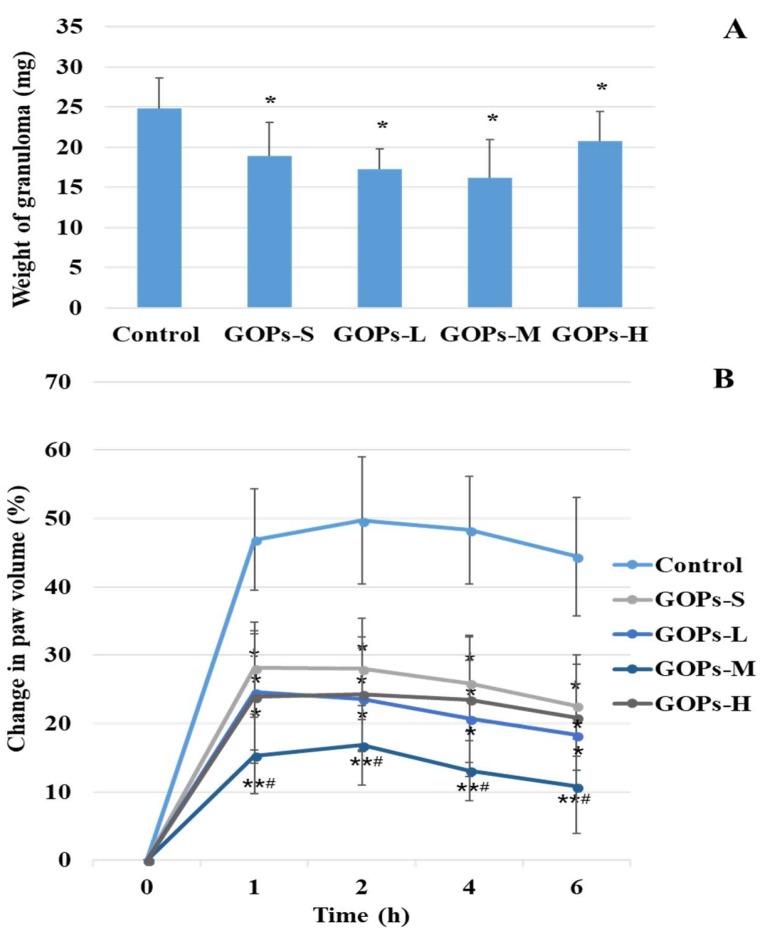
Effect of GOPs on cotton pellet-induced granuloma (**A**) and dextran-induced paw edema (**B**) in rats. Data are expressed as means ± SD and analysis via the one-way analysis of the variance test. *n* = 10/group. * *p* < 0.05, ** *p* < 0.01 compared with the control. # *p* < 0.05 compared with the GOPs-S group. GOPs-S, 62.5 mg/kg*bw ginseng oligopepides treated group. GOPs-L, 125 mg/kg*bw ginseng oligopepides treated group. GOPs-M, 250 mg/kg*bw ginseng oligopepides treated group. GOPs-H, 500 mg/kg*bw ginseng oligopepides treated group.

Figure 1A summarized the effect of GOPs on chronic inflammation in rats. The doses of GOPs groups (GOPs-S, GOPs-L, GOPs-M, and GOPs-H) were 62.5, 125, 250, and 500 mg/kg*bw, respectively. The weight of granuloma in GOP groups present a value lower than the control with 23.79%, 30.65%, 34.68%, and 16.53% reduction, respectively. It was indicated that GOPs had a significant suppression activity on granuloma (*p* < 0.05 for all GOPs groups).

### 2.2. Effects of GOPs on Dextran Sulphate-Induced Paw Edema

Compared with the control, treatment with GOPs caused a dose-dependent significant inhibition of swelling caused by the dextran (*p* < 0.05 for GOPs-S, GOPs-L and GOPs-H, *p* < 0.01 for GOPs-M, shown Figure 1B). Compared with the GOPs-S group, the change in paw volume were significantly decreased in GOPs-M (*p* < 0.05) and GOPs at the dose of 250 mg/kg*bw (GOPs-M) showed the best ability to inhibit the extent of 72.88% 4 h after dextran injection compared with the control.

### 2.3. Effects of GOPs on Inflammatory Parameters in Serum

For the granuloma model, the levels of serum NO was detected. The levels were 36.2 ± 3.1 μmol/L (control), 41.3 ± 7.9 μmol/L (GOPs-S), 32.7 ± 2.3 (GOPs-L), 31.5 ± 2.5 (GOPs-M), and 21.4 ± 2.1 μmol/L (GOPs-H), respectively. Compared with the control, the levels of NO were significantly decreased in GOP-L, GOP-M, and the GOP-H group (*p* < 0.05). It is interesting to note that the NO level was slightly increased in GOPs-S rats without significance (*p* > 0.05).

As shown in Table 1, high levels of serum TNF-α and IL-1β were detected in control rats. However, the levels of the tumor necrosis factor-α (TNF-α) and interleukin IL-β (IL-1β) in GOP groups were significantly reduced (*p* < 0.05). Particularly, compared with the GOPs-S, the levels remarkably decreased in GOPs-L and GOPs-M groups. It was revealed that the level of interleukin IL-10 (IL-10) in the control were significantly lower than that in GOPs-treated rats (*p* < 0.05). Similarly, IL-10 is significantly lower than in the GOPs-L group.

### 2.4. Effects of GOPs on Inflammatory Parameters in Paw Tissue

As shown in Table 2, high levels of TNF-α, prostaglandin E2 (PGE_2_), Leukotriene D4 (LTD_4_), and the platelet activating factor (RAF) were detected in control rats. The levels of TNF-α, LTD_4_, and RAF in GOP groups were significantly reduced while the level of PGE_2_ was significantly increased (*p* < 0.05). Particularly, compared with the GOPs-S, the levels remarkably changed in GOPs-L and GOPs-M groups.

### 2.5. Effects of GOPs on mRNA Expression of p38 MAPK and NF-κB p65 in Paw Tissue

**Figure 2:**
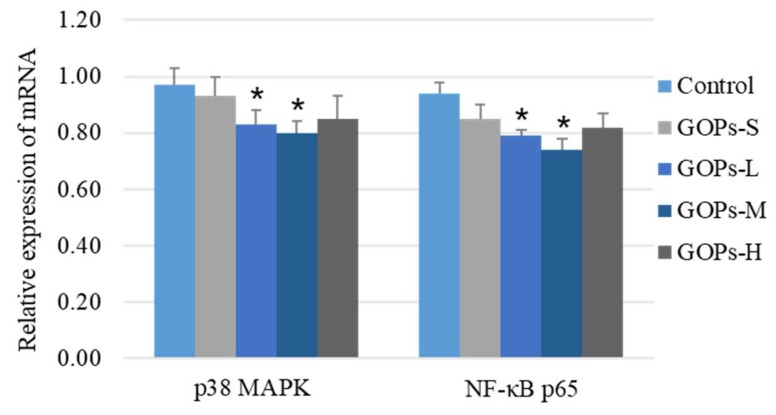
Effect of GOPs on the mRNA expression of p38 MAPK and NF-κB p65 in paw tissue of rats by real-time PCR analysis. β-actin mRNA levels were used as a control. Data are expressed as means ± SD and analysis via the one-way analysis of the variance test. *n* = 8/group. * *p* < 0.05 compared with the control. p38 MAPK, p38 mitogen activated protein kinase. NF-κB p65, Nuclear factor-κB p65. GOPs-S, 62.5 mg/kg*bw ginseng oligopepides treated group. GOPs-L, 125 mg/kg*bw ginseng oligopepides treated group. GOPs-M, 250 mg/kg*bw ginseng oligopepides treated group. GOPs-H, 500 mg/kg*bw ginseng oligopepides treated group.

As shown in Figure 2, compared with the control, the mRNA expression of p38 MAPK and NF-κB p65 was markedly decreased in GOPs-L and GOPs-M groups (*p* < 0.05). 

## 3. Discussion

Recently, food-derived peptides have been considered one of the most effective alternatives to drugs, as anti-inflammatory peptides. In this study, we evaluated the positive effects of GOPs on anti-inflammatory by down regulating cytokines and inhibiting gene expression related to inflammation. The results of our study showed that GOPs could inhibit development of paw edema over a period of 6 h following dextran injection, especially for the first hour. It indicated the beneficial effect of GOPs on acute inflammation. As for chronic inflammation, we found that GOPs could ameliorate granuloma induced by cotton pellet-induced factors as well as reduced inflammatory parameters in serum. These findings were generating important information for health.

The dextran-induced paw edema model was used to study the acute anti-inflammatory effect of GOPs. As an indicator of edema induced by dextran, the volumes of paw were significantly suppressed by GOPs. As a potent osmotic agent, dextran induces an anaphylactic reaction [17,18]. Similar to other inflammatory factors, the inflammatory response, triggered by dextran, is characterized into two phases. For the first phase (0–1 h), it is characterized by extravasation and edema formation as a result of histamine and causes a significant increase in vascular permeability and blood flow to the inflammatory site. For the second phase (1–6 h), it is marked by serotonin release from the mast cells and featured by the release of free radicals, bradykinin, PGE_2_, LTD_4_, NO, and cytokines (IL-1β, TNF-α, IL-10), which was derived from neutrophil infiltration [19,20]. The cyclooxygenase-2 (COX-2) enzyme is significantly expressed in the treated paw. In addition, it is contributed to vasodilatation and the production of a pro-inflammatory mediator (PGs and LTs), which results in the symptom of fever and pain [21]. Our findings demonstrated that GOPs could inhibit the edema formation, which is related to the decreasing levels of LTD_4_ and RAF that contributed to vascular changes of edema. PGE_2_ is one of the most abundant prostaglandins and it involved all the processes of the inflammatory classic signs. PGE_2_-mediated arterial dilatation and enhanced vascular penetrability, which enhanced blood flow in the inflamed tissue. Lastly, it resulted in redness and edema as a clinical sign. It is interesting to note that the PGE_2_ level was increased in GOPs-treated rats, which was in contrast with the reduction of edema. It is indicated that GOPs were not able to suppress PGE_2_-induced inflammation, and possible via regulating hypersensitivity mediated by LTD_4_. Cytokines play a crucial role in various kinds of chronic disease, which involve both the generation and maintenance [22,23,24]. Even though, compared with the second phase, the changes of paw volume mainly occurred in the first hour, our results indicated that GOPs have a beneficial effect in the second phase. Thus, it was studied whether the anti-inflammatory activities of GOPs was related to the suppression of TNF-α. Similarly, our data showed that GOPs considerably reduced the TNF-α levels into the dextran-injected paw tissues. MAPK and NF-κB, as transcription factors, play critical roles in proliferation and apoptosis [21,22,23,24]. As for acute inflammatory, we mainly test the signals at the transcriptional level by PCR. Our results demonstrated that GOPs inhibit inflammatory effects by regulating gene (MAPK and NF-κB) expression responsible for inflammation. In short, the anti-edematogenic effect of GOPs might concern cellular membrane stabilization of mast cells as well as the inhibition of the release and action of cytokines.

Furthermore, the cotton pellet-induced granuloma model is widespread and used to assess the transudative and proliferative inflammation due to its similarity to human chronic inflammatory [25]. As an indicator of macrophage activation, dysfunction, and invasion, granuloma formation was measured according to the increase of implanted pellet [26,27,28]. In the present study, GOPs exhibited dose-dependent suppression activity on granuloma, which suggested that GOPs benefit the inhibition of chronic inflammatory. Decreasing the size of granuloma tissue, it is well correlated with decreased pro-inflammatory cytokines levels (e.g., TNF-α, and IL-1β), as well as an increased level of IL-10 [29,30,31]. Previous studies demonstrate that oral administration of GOPs increased the activity of NK cell, macrophage phagocytosis, and the Th cells stimulation, which is followed by cytokine secretion and antibody production [13,15]. Consistent with the reduction effects of GOPs on TNF-α and free radicals [13,14], this current study showed GOPs significantly downregulated the expression of TNF-α and IL-1β as well as increased IL-10 expression in serum. NO, as an intracellular messenger, is critically important in a wide range of pathological and inflammatory conditions, which was macrophages-activated [32,33,34]. Along with the inhibition of cytokines, GOPs suppressed NO production in serum.

There are certain limitations included in this study, which can be topics for further study. For instance, (1) there is no positive control group and (2) we did not do the histological observation since no paws samples were fixed.

## 4. Materials and Methods 

### 4.1. Materials and Animals

Jilin Taigu Biological Engineering Co., Ltd. (Jilin, China) provided the GOPs substances. It was derived from the roots of *Panax ginseng* C. A. Meyer planted in Jilin province, China. GOP powders were obtained from ginseng roots through multiple processes including boiled, centrifuged, centration, purification, and spray drying, which was described in a previous study [13,14,15]. Dextran sulphate powder (40,000 Da, Sigma-Aldrich, St. Louis, MO, USA). All other reagents were of an analytical grade. As the basal diet, HFK Bioscience Co. Ltd. (Beijing, China) produced the AIN-93G rodent diet. 

Furthermore, 100 male S-D rats (4 weeks old, 180–200 g) were provided by the Animal Service of Health Science Center, Peking University, Beijing. Rats were housed two per plastic cages in a SPF (Specific Pathogen Free) filter-protected air-conditioned room with controlled temperature (21–25 °C), relative air humidity (50 ± 5%), and 12-hour light/dark cycles (light on 07:30–19:30 hours). All animals were handled in accordance with the guidelines of the Principle of Laboratory Animal Care (NIH publication No. 85–23, revised 1985) of the Peking University Animal Research Committee (www.lab.pku.edu.cn, Ethical approval code: LA2015081, February 2015).

After acclimatization for one week, the rats were randomly divided into five groups (Rats, *n* = 20/group): control group (10 mL/kg distilled water), and four GOPs intervention groups which were designated as two low-dose (GOPs-S, GOPs-L), one medium-dose (GOPs-M), and one high-dose (GOPs-H). GOPs were administered to the rats of the four GOPs groups at 62.5, 125, 250, and 500 mg/kg*bw, respectively. The doses refer to the previous study in our lab [13,14,15]. During the experimental period, all rats were administrated by gavage for 30 days as well as fed freely with a basal diet and tap water.

### 4.2. Cotton Pellet-Induced Granuloma

The cotton pellet-induced granuloma experiment was used as a chronic inflammation model. On Day 22, the rats (*n* = 10/group) were anaesthetized with ether, and incision was made on the lumbar region [27]. By blunted forceps, a subcutaneous tunnel was made and a sterilized cotton pellet (100 ± 1 mg) was inserted in the groin area. At the end of the experiment, the animals were anaesthetized again and then sacrificed. The cotton pellets were surgically removed without extraneous tissues and dried at 60 °C until the weight remained constant and the net dry weight was calculated. Blood was obtained from the femoral artery and serum was separated (3000 g for 20 min at 4 °C) for biochemical assays.

### 4.3. Dextran Sulphate-Induced Paw Oedema 

As the acute inflammation model, dextran sulphate-induced paw edema experiment was carried. The paw edema of rats (*n* = 10/group) was induced by a method previously described on Day 30 [35]. Furthermore, 0.1 mL of 1% (*w*/*v*) dextran sulphate dissolved in saline solution was injected into the sub-plantar region of the right hind paw of rats. The extent of swelling of the paw was quantitated by measuring the volume of the paws before edema induction and then at 1, 2, 4, and 6 h with intervals post-injection, by a YLS-7B paw volume admeasuring apparatus (Shandong Medicine Institute, Shandong, China). Increase in paw volume was expressed as the mean percentage change of the paw volume using the formula below.

$$\%\;change\;in\;paw\;volume=\left(\frac{PT_{0}-PT_{t}}{PT_{0}}\right)\times 100$$

They were anesthetized by CO_2_ inhalation and then sacrificed. The paws were frozen in liquid nitrogen and then saved in −80 °C for future experiments.

### 4.4. Biochemical Assay

The levels of TNF-α, IL-1β, IL-10, and NO in mice serum, similarly to TNF-α, LTD_4_, RAF, and PGE_2_ in paw muscles tissue, which were measured by an enzyme linked immunosorbent assay (ELISA), according to the kit’s instructions. All detection kits were provided by the Beyotime Institute of Biotechnology (Beijing, China). 

### 4.5. Quantitative Real-Time PCR and Analyses

RNA was extracted from isolated paw tissue of rats form Dextran sulphate-induced paw edema experimental using Trizol-A+ reagent. Furthermore, RT reactions were performed with total RNA, according to the FastQuant RT Kit (TIANGEN, Beijing, China). Real-time reverse transcription-PCR was performed. ABI 7300 real-time PCR detection system was used to detect the RNA expression of target genes with especic primers: p38 mitogen activated protein kinase (p38 MAPK), Forward 5′-ATAATGCGTCTGACGGGGAC-3′ and Reverse 5′-GGGTCGTGGTACTGAGCAAA-3′; Nuclear factor-κB p65 (NF-κB p65), Forward 5′-TGAGCGTAGGTGATGAGTGC-3′ and Reverse 5′- GCCTGGTCCCGTGAAATACA-3′; β-actin, Forward 5′-CGGTTGGCCTTAGGGTTCAGG-3′ and Reverse 5′- GTGGGCCGCTCTAGGCACCA-3′. The product was amplified in a reaction volume of 25 μL including 12.5 μL 2 × SuperReal PreMix, 1 × U ExTaq DNA polymerase and 0.75 μL of each primer (10 μM). PCRs were performed for 40 cycles at 95 °C for 10 s, 58 °C for 20 s, and 72 °C for 30 s. PCR was performed in triplicate for each sample. The relative amount of mRNAs was normalized against β-actin mRNA, and the fold change for each mRNA was calculated by the 2^−^^ΔΔCt^ method [36].

### 4.6. Statistical Analysis

Statistical analyses were performed with SPSS software (version 19.0, SPSS Inc., Chicago, IL, USA). Variances in the measurement data were checked for homogeneity. Then the one-way analysis of variance test and LSD methods were used. A value of *p* < 0.05 was considered significant. 

## 5. Conclusions 

Taken together, referred to our results, GOPs supplement showed a positive effect on both acute and chronic inflammation, which was possibly related to inflammatory mediators’ modulation and inhibition of inflammation genes’ expression. Further research is necessary to study the dose and mechanisms of anti-inflammation.

## Figures and Tables

**Table 1:** Effect of GOPs on inflammatory parameters in serum of granuloma rats.

	TNF-α (ng/L)	IL-1β (ng/L)	IL-10 (ng/L)
Control	309.9 ± 16.1	46.2 ± 2.6	30.4 ± 3.1
GOPs-S	257.2 ± 18.3 *	32.9 ± 1.3 *	38.6 ± 5.3 *
GOPs-L	237.7 ± 17.8 *^,#^	26.8 ± 1.9 *^,#^	56.2 ± 2.9 *^,#^
GOPs-M	202.4 ± 17.3 *^,#^	21.4 ± 2.4 *^,#^	68.4 ± 3.9 *^,^^#^
GOPs-H	307.5 ± 18.9	30.4 ± 2.9 *	73.4 ± 4.7 *^,#^

Data are expressed as means *±* SD and analysis via the one-way analysis of the variance test. *n* = 10/group. TNF-α, tumor necrosis factor-α, IL-1β, interleukin IL-β, IL-10, interleukin IL-10, GOPs, ginseng oligopeptides. * *p* < 0.05 compared with the control. # *p* < 0.05 compared with the GOPs-S group. GOPs-S, 62.5 mg/kg*bw ginseng oligopepides treated group. GOPs-L, 125 mg/kg*bw ginseng oligopepides treated group. GOPs-M, 250 mg/kg*bw ginseng oligopepides treated group. GOPs-H, 500 mg/kg*bw ginseng oligopepides treated group.

**Table 2:** Effect of GOPs on inflammatory parameters in paw tissue.

	TNF-α (pg/mg)	PGE_2_ (pg/mg)	LTD_4_ (pg/mg)	RAF (pg/mg)
Control	789.4 ± 58.2	356.2 ± 40.1	236.6 ± 17.9	33.7 ± 6.4
GOPs-S	743.8 ± 92.7	379.0 ± 30.2 *	214.3 ± 22.3 *	22.8 ± 6.3 *
GOPs-L	636.3 ± 112.9 *^,#^	402.1 ± 34.2 *^,#^	213.5 ± 19.2 *	17.0 ± 5.1 *^,#^
GOPs-M	617.3 ± 105.4 *^,#^	443.2 ± 46.8 *^,#^	182.5 ± 10.5 *^,#^	14.0 ± 4.9 *^,#^
GOPs-H	738.0 ± 108.8	366.8 ± 39.2 *	221.2 ± 34.4	23.9 ± 2.5 *

Data are expressed as means ± SD and analysis via the one-way analysis of the variance test. *n* = 10/group. TNF-α, tumor necrosis factor-α. LTD_4_, leukotrienes D_4_. RAF, platelet activating factor. PGE_2_, prostaglandin E_2_. GOPs, ginseng oligopeptides. * *p* < 0.05 compared with the control. # *p* < 0.05 compared with the GOPs-S group. GOPs-S, 62.5 mg/kg*bw ginseng oligopepides treated group. GOPs-L, 125 mg/kg*bw ginseng oligopepides treated group. GOPs-M, 250 mg/kg*bw ginseng oligopepides treated group. GOPs-H, 500 mg/kg*bw ginseng oligopepides treated group.
